# *Vigna radiata* (L.) R. Wilczek Extract Inhibits Influenza A Virus by Targeting Viral Attachment, Penetration, Assembly, and Release

**DOI:** 10.3389/fphar.2020.584973

**Published:** 2020-11-26

**Authors:** Chieh-Wen Lo, Chia-Chen Pi, You-Ting Chen, Hui-Wen Chen

**Affiliations:** ^1^ Department of Veterinary Medicine, National Taiwan University, Taipei, Taiwan; ^2^ King’s Ground Biotech Co., Ltd., Pingtung, Taiwan

**Keywords:** *Vigna radiata* extract, mung bean, functional foods, antiviral activity, influenza virus

## Abstract

*Vigna radiata* (L.) R. Wilczek (mung bean) is a Chinese functional food with antioxidant, antimicrobial and anti-inflammatory activities. However, little is known about its antiviral activity. We aimed to investigate the antiviral activity and mechanisms of action of *Vigna radiata* extract (VRE) against influenza virus. HPLC was conducted to analyze the components of the VRE. The anti-influenza viral activity of VRE in Mardin-Darby canine kidney (MDCK) cells was evaluated by virus titration assays, hemagglutination assays, quantitative RT-PCR assays, cellular *α*-glucosidase activity assays and neuraminidase activity assays. Chromatographic profiling analysis identified two major flavonoids, vitexin and isovitexin, in the ethanol extract of *Vigna radiata*. Through *in vitro* studies, we showed that VRE, at concentrations up to 2,000 μg/ml, exhibited no cytotoxicity in MDCK cells. VRE protected cells from influenza virus-induced cytopathic effects and significantly inhibited viral replication in a concentration-dependent manner. A detailed time-of-addition assay revealed that VRE may act on both the early and late stages of the viral life cycle. We demonstrated that 1) VRE inhibits virus entry by directly blocking the HA protein of influenza virus; 2) VRE inhibits virus entry by directly binding to cellular receptors; 3) VRE inhibits virus penetration; 4) VRE inhibits virus assembly by blocking cellular *α*-glucosidase activity, thus reducing HA protein trafficking to the cell surface; and 5) VRE inhibits virus release by inhibiting viral neuraminidase activity. In summary, *Vigna radiata* extract potently interferes with two different subtypes of influenza viruses at multiple steps during the infectious cycle, demonstrating its broad-spectrum potential as an anti-influenza preventive and therapeutic agent. Continued development of *Vigna radiata*-derived products into antiviral therapeutics is warranted.

## Introduction

Influenza A virus infection is a zoonotic infectious disease that continues to pose a pandemic threat worldwide. Epidemiological studies from 2011 to 2018 indicate that influenza A/H3N2 (39.2%) and A/H1N1 (28.3%) are the dominant strains circulating in Asian countries (Young and Chen, 2020). Influenza A virus contains eight negative-sense single-stranded viral RNA (vRNA) segments, encoding for more than 10 viral proteins. For the viral envelope-associated proteins, the hemagglutinin (HA) interacts with the host cell receptor, and the neuraminidase (NA) plays a role in viral release. The matrix protein 1 forms a coat inside the viral envelope, and the matrix protein 2 is a proton-selective ion channel protein. For the internal proteins, polymerase basic protein 1 and 2 (PB1, PB2), polymerase acidic protein (PA) and nucleoprotein (NP) are members of the viral ribonucleoprotein (vRNP), responsible for vRNA replication. Non-structural protein 1 and 2 (NS1, NS2) have roles in host immunity modulation and vRNP transportation, respectively (Bouvier and Palese, 2008). The influenza A virus life cycle begins with the attachment between viral HA protein and host sialic acid. After that, the virus penetrates into the host cell through cellular endocytic machinery. Followed by endosomal acidification, the vRNP is released from the endosome into the cytosol. The vRNP then traffics into the nucleus and initiates the vRNA replication. The newly synthesized RNA is transported back to the cytosol for viral protein translation. After post-translational glycosylation of viral HA and NA, these proteins traffic to the cell membrane together with newly synthesized vRNP to form viral particles. Finally, the viral NA with sialidase activity cuts the linkage of viral HA and host sialic acid, leading to the release of viral particles (Dou et al., 2018).

Due to its high mutation and genomic recombination rates, new strains of influenza A virus occur frequently, and some of these strains are associated with high morbidity and mortality. In addition to the seasonal H1N1 or H3N2 strains, highly pathogenic avian influenza H5N1 viruses with human adaptive mutations in the PB2, PA, NP and M genes were found in 18 human infected cases in Hong Kong and led to one third of the infected people died (Zhou et al., 1999). Although a vaccine for seasonal influenza is available, the protective effect varies annually depending on the coverage of the vaccine formulation (Wei et al., 2020). A small number of antiviral drugs have been approved, including oseltamivir, zanamivir, and peramivir, which target viral NA to inhibit virus release from host cells (Moscona, 2008). However, drug-resistant strains have already been reported (Lampejo, 2020). Recently, baloxavir, a novel viral PA protein inhibitor, was approved in 2018 (Abraham et al., 2020). However, strains with reduced susceptibility have already emerged (Sato et al., 2020). Thus, there is an urgent need for development of new antiviral drugs.

Herbal medicines have been proven to be applicable to combat various viral infectious diseases (Li and Peng, 2013). In contrast to single-compound-based medicines, one of the advantages of herbal medicines is that they contain several active components that may simultaneously act on viruses or hosts, interfering with the viral life cycle at different stages. For example, the extract of *Houttuynia cordata* has multiple inhibitory effects on SARS-CoV by enhancing host IL-2 and IL-10 secretion and inhibiting the viral protease and RNA-dependent RNA polymerase (Lau et al., 2008). Therefore, herbal medicine-based treatment is a prominent strategy to protect people from drug-resistant viruses. Mung bean (*Vigna radiata* (L.) R. Wilczek) is one of the most important crops grown in countries of south, east and southeast Asia (Nair et al., 2013), and it is commonly used as a folk medicine in Taiwan and China due to its anti-inflammatory effects and ability to relieve heat stress (Cao et al., 2011; Zhang et al., 2013). The composition of mung bean has been reported (Ganesan and Xu, 2018). The crude extract of mung bean has been shown to have antioxidant, antidiabetic and immunoregulatory activities (Randhir and Shetty, 2007; Bai et al., 2016; Hashiguchi et al., 2017). Furthermore, extracts of mung bean sprouts exhibit the ability to protect against respiratory syncytial virus and herpes simplex virus 1 (Hafidh et al., 2015). However, whether mung bean has an inhibitory effect on influenza virus has not yet been investigated. In this study, we demonstrated that mung bean exerts anti-influenza A virus effects by blocking viral attachment, entry, glycoprotein membrane trafficking and neuraminidase activity, with no cytotoxic effects.

## Materials and Methods

### Materials and Preparation of *Vigna radiata* Extract

*Vigna radiata* (L.) R. Wilczek (mung bean) was provided by King’s Ground Biotech Co., Ltd. (Pingtung, Taiwan) with a batch number NTU01. *Vigna radiata* extract (VRE) was prepared from the seed coats of mung bean via physical, chemical and biological processes. After mixing with organic acids under high temperature and pressure, the seed coats of mung beans were dried by hot air and ground to a powder. The VRE product contained 13% crude protein, 70% carbohydrate, and 4% ash. The VRE powder was then extracted with 95% ethanol (1:10 w/v) and sonicated for 1 h. The filtered solvent of the extract was concentrated using a rotary evaporator (Eyela, Japan). The recovery of the dried extract was approximately 15–25% (w/w). The dried extract was stored at −20°C until use.

### High Performance Liquid Chromatography Analysis

All solvents used were HPLC grade, and all reagents were analytical grade. The analysis was performed using a high-performance liquid chromatography system (Hitachi D2000), which consisted of an L-2130 HTA Pump, an L-2200 autosampler, and an L-2455 diode array detector. The extracted compound was separated using an Inertsil ODS-2 C18 column (250 mm × 4.6 mm, 5 μm) with a gradient mobile phase of 0.3% *ortho*-phosphoric acid (Scharlab) in ddH_2_O (solvent A) and acetonitrile (Fisher Chemical) (solvent B). HPLC was performed at a flow rate of 0.8 ml/min with detection at 220 nm. The column temperature was maintained at 30°C. All samples were diluted in methanol (Honeywell) before analysis. Chromatographic peaks were identified on the basis of retention time.

### Cells and Viruses

Mardin-Darby canine kidney (MDCK) cells (ATCC #CCL-34) were maintained in DMEM supplemented with 10% FBS and 1% penicillin/streptomycin/amphotericin B and cultured at 37°C with 5% CO_2_. For influenza virus infection in MDCK cells, infection medium (RPMI containing 0.75% BSA, 0.05 mM 2-mercaptoethanol, 1% PSA, and 2 μg/ml L-1-*p*-tosylamino-2-phenylethyl chloromethyl ketone-treated trypsin) was used. All the reagents for cell culture were purchased from Invitrogen. Influenza A viruses, A/Puerto Rico/8/1934 (PR8-H1N1) and A/Chicken/Taiwan/3937/2012 (3937-H6N1) (Zhu et al., 2020), were propagated in MDCK cells and titrated with as previous described (Hu et al., 2018). All types of influenza viruses were propagated in MDCK cells. Viruses were titrated by 50% tissue culture infective dose (TCID_50_) assays, hemagglutination test and quantitative RT-PCR (qRT-PCR) as previously described (Chen et al., 2013). For the TCID_50_ assay, in brief, MDCK cells in the 96 well plate (2 × 10^4^ cells per well) were infected with 100 μl of 10-fold serially diluted virus-containing sample and incubated at 37°C for 3 days. Following incubation, viral infection in each well was determined by a hemagglutination assay using 1% chicken red blood cells. Viral titers were calculated by the Reed-Muench method (Reed and Muench, 1938).

### Cytotoxicity Test

The cytotoxicity of VRE in MDCK cells was examined. Briefly, 2 × 10^4^ cells were seeded in 96-well plates overnight. Then, VRE serially diluted in medium was applied to the cells and incubated for 24, 48, or 72 h. The culture medium was removed, and 100 μl of 0.5 mg/ml 3-(4,5-dimethylthiazol-2-yl)-2,5-diphenyltetrazolium bromide (MTT) solution was added, followed by incubation at 37°C for 3 h. The supernatants were removed, and 100 μl of dimethyl sulfoxide was added to each well. The absorbance value of each well was determined spectrophotometrically at 570 nm in a microplate reader (Synergy H1, BioTek, Winooski, VT, United States).

### Antiviral Activity Test

MDCK cells (2 × 10^5^) were seeded in 24-well plates overnight. PR8-H1N1 (multiplicity of infection, MOI = 0.1) was treated with serially diluted VRE at 37°C for 1 h. At the same time, MDCK cells were incubated with serially diluted VRE in medium at 37°C for 1 h. After incubation, the VRE-virus mixture was used to infect cells for 1 h. The cells were washed after supernatant removal and then incubated with serially diluted VRE for 24 h. The supernatants were collected for the TCID_50_ assay to determine the viral titer. The viral titers of each group relative to the vehicle control were calculated.

### Time-of-Addition Assay

MDCK cells (2 × 10^5^) were seeded in 24-well plates overnight. PR8-H1N1 (MOI = 2) was incubated with 2,000 μg/ml VRE under six different conditions. 1) For the “pretreated virus” group, the virus was incubated with VRE in medium for 1 h before infecting MDCK cells for 1 h. After infection, the medium containing virus and VRE was removed, fresh medium was added, and the cells were incubated for an additional 11 h. 2) For the “pretreated cell” group, MDCK cells were incubated with VRE for 1 h before infection. One hour later, the virus was added and allowed to infect the cells for another 1 h. After infection, the VRE- and virus-containing medium was changed to fresh medium, and the cells were incubated for an additional 11 h. 3) For the “pretreated cell + wash” group, MDCK cells were incubated with VRE for 1 h, and the medium was replaced with fresh medium before virus infection. After 1 h of virus infection, the medium that contained the virus was removed, fresh medium was added, and the cells were incubated for an additional 11 h. 4) For the “treated infection + wash” group, virus-infected MDCK cells were treated with VRE for 1 h. After 1 h of virus infection, the medium was removed, fresh medium was added, and the cells were incubated for an additional 11 h. 5) For the “after infection” group, MDCK cells were treated with VRE after virus infection for 11 h. 6) For “all”, the virus was preincubated with VRE in medium for 1 h. At the same time, MDCK cells were preincubated with VRE for 1 h. After incubation, the VRE-virus mixture was used to infect cells for 1 h. The cells were washed after supernatant removal and then incubated with VRE for 11 h. The supernatants were collected for the TCID_50_ assay to determine the viral titers. To further dissect the time course of VRE’s mechanism of action, another time-of-addition assay was also performed. VRE was added at 0, 2, 4, 6, and 8 h post virus infection. The supernatant was collected at 12 h post infection for the TCID_50_ assay to determine the viral titer. Viral RNA in the cells was extracted and detected by qRT-PCR (Chen et al., 2013).

### Hemagglutination Assay Using *Vigna radiata* Extract-Treated Red Blood Cells or Viruses

Chicken RBCs (1%) were mixed with various concentrations of VRE in a U-bottomed microplate and incubated at room temperature (RT) for 30 min. The hemagglutination activity of VRE was observed by eye. To determine VRE binding to the surface receptor of RBCs and its inhibition of virus-induced hemagglutination, 500 μl of 1% RBCs were treated with various concentrations (0, 125, 500, and 2,000 μg/ml) of VRE at RT for 30 min. The unbound VRE was removed by centrifugation at 1,000 ×g for 5 min, and the treated RBC pellet was washed and resuspended in 500 μl of PBS. Next, 2,000 hemagglutination unit (HAU) of PR8-H1N1 virus was added to treated RBCs and incubated at RT for 30 min. Then, the RBC pellet was washed and resuspended in 100 μl of PBS. The amount of RBC-bound virus was determined by qRT-PCR. To determine VRE binding to the viral surface and its inhibition of virus-induced hemagglutination, 2,000 HAU of PR8-H1N1 virus was treated with various concentrations (0, 125, 500, and 2,000 μg/ml) of VRE or 2 μg of polyclonal antibody against H1N1 hemagglutinin (SinoBiological #11684-T56) at RT for 30 min. Next, 500 µl of 1% RBCs was added to the treated virus solution and incubated at RT for 30 min, and then the RBC pellet was washed and resuspended in 100 μl of PBS. The amount of RBC-bound virus was determined by qRT-PCR.

### Penetration Inhibition Assay

MDCK cells (2 × 10^5^) were seeded in 12-well plates overnight before being infected with PR8-H1N1 (MOI = 2) at 4°C for 1 h. After virus binding, VRE was applied to cells for a 1 h incubation at 37°C. Unpenetrated viruses were inactivated by incubation with PBS (pH 2) for 1 min and then neutralized with PBS (pH 11). After incubation for an additional 6 h, the cells were washed and fixed with 4% paraformaldehyde (Sigma) for 20 min at room temperature. The cells were permeabilized with 0.3% Triton X-100 (Sigma) in 0.1% BSA for 30 min and then blocked with 1% BSA for 30 min at room temperature. The primary antibody, anti-NP (1:1,000, HyTest #3IN5), was diluted in 1% BSA and incubated with cells overnight at 4°C. The secondary antibody, FITC-conjugated goat anti-mouse IgG (1:1,000, Jackson ImmunoResearch), was diluted with 1% BSA and incubated with the cells for 1 h at room temperature. Nuclei were stained with DAPI (1:1,000, Invitrogen) in PBS for 10 min at room temperature. Fluorescence images were recorded with a microscope (Olympus IX-83), and the fluorescence signal was measured using a microplate reader (Synergy H1, BioTek). The NP expression levels relative to the nuclear signal were determined.

### *α*-Glucosidase Inhibition Assay

The *α*-glucosidase inhibition assay was performed according to a previous study (Pullela et al., 2006) with some modifications. Briefly, 0.8 μl of various concentrations of VRE was mixed with 69.2 μl of phosphate buffer (100 mM, pH 6.8) and 10 μl of *α*-glucosidase (1 U/ml, Sigma) and incubated for 15 min at 37°C. After incubation, 20 μl of substrate, *p*-nitrophenyl-*α*-d-glucopyranoside (5 mM, Sigma), was added and incubated for 20 min at 37°C. The reaction was stopped by adding 50 μl of Na_2_CO_3_ (0.1 M). The absorbance at 450 nm was recorded before and after substrate addition, and an increase in the absorbance was observed. Relative *α*-glucosidase inhibition was determined as a percentage of the control.

### Detection of Viral Hemagglutinin Surface Expression

The HA trafficking inhibition assay was performed as previously described (Saito and Yamaguchi, 2000) with some modifications. Briefly, 2 × 10^5^ MDCK cells were seeded in 12-well plates overnight before being infected with PR8-H1N1 (MOI = 10) for 1 h. After the virus was removed, the cells were treated with various concentrations of VRE for 2–5 h post infection. The virus signal was then detected with immunofluorescence. For HA staining in the entire cell (surface and intracellular HA protein), the cells were fixed with 4% paraformaldehyde and then permeabilized with 0.3% Triton X-100 in 0.1% BSA. For HA staining on the cell surface, the cells were fixed with 4% paraformaldehyde before staining without permeabilization. The cells were then blocked with 1% BSA for 30 min at room temperature. The primary antibody anti-HA (1:500, SinoBiological #11684-T56) was diluted in 1% BSA and incubated with cells overnight at 4°C. The secondary antibody, FITC-conjugated goat anti-rabbit IgG (1:1,000, Jackson ImmunoResearch), was diluted with 1% BSA and incubated with cells for 1 h at room temperature. Nuclei were stained with DAPI (1:1,000, Invitrogen) in PBS for 10 min at room temperature. Fluorescence images were recorded with a microscope (Olympus IX-83), and the fluorescence signal was measured using a microplate reader (Synergy H1, BioTek). The HA expression levels relative to the nuclear signal were determined from the two different types of staining methods.

### Neuraminidase Inhibition Assay

The neuraminidases from mammalian influenza virus PR8-H1N1 and avian influenza 3937-H6N1 were utilized to investigate the inhibition ability of VRE. The neuraminidase inhibition assay was performed according to Zhu et al. (2015) with some modifications. Briefly, 1 μl of various concentrations of VRE or oseltamivir (Sigma) was mixed with 25 μl of virus (32 HAU of 3937-H6N1 virus or PR8-H1N1 virus) and 1× assay buffer (33 mM MES, 4 mM CaCl_2_, pH 6.5) to a total of 50 μl and then incubated for 20 min at 37°C. Fifty microliters of fluorogenic substrate (50 μM 4-methylumbelliferyl-N-acetylneuraminic acid, Sigma) was added and incubated for 60 min at 37°C. The reaction was then terminated by adding 100 μl of 0.2 M Na_2_CO_3_. Fluorescence was measured with an excitation wavelength of 355 nm and an emission wavelength of 460 nm. Relative fluorescence units were obtained by subtracting the background value.

### Transmission Electron Microscopy

A total of 10^6^ MDCK cells were seeded onto a sterile plastic sheet in a 6-well plate overnight before infection with PR8-H1N1 (MOI = 5) for 1 h. The cells were treated with VRE (2,000 μg/ml) or oseltamivir (5 μg/ml) for 6–12 h post infection. The cells were then washed and fixed with 2.5% glutaraldehyde for TEM imaging. Standard operating procedures were performed for imaging via transmission electron microscopy (FEI Tecnai TF20).

### Statistical Analysis

Data were analyzed by an unpaired *t* test or ANOVA with Dunnett’s multiple comparison test using Prism (GraphPad, San Diego, CA, United States). *p* Values smaller than 0.05 were considered significant.

## Results

### HPLC Profile of *Vigna radiata* Extract

**FIGURE 1:**
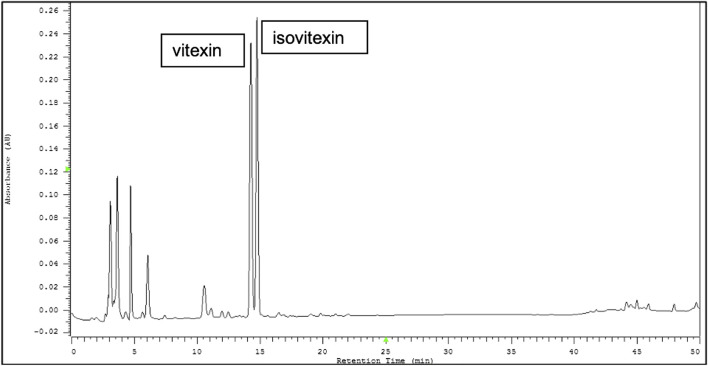
Representative HPLC chromatograms from the seed coats of *Vigna radiata* (L.) R. Wilczek (mung bean). Vitexin and isovitexin were found to be two major components of *Vigna radiata* extract (VRE).

Representative HPLC chromatograms from the seed coats of *Vigna radiata* (mung bean) are shown in Figure 1. Vitexin and isovitexin were found to be two major ingredients in VRE. The active compounds contain 22.153 mg/g vitexin and 24.171 mg/g isovitexin.

### *Vigna radiata* Extract Shows No Cytotoxicity in Mardin-Darby Canine Kidney Cells

**FIGURE 2:**
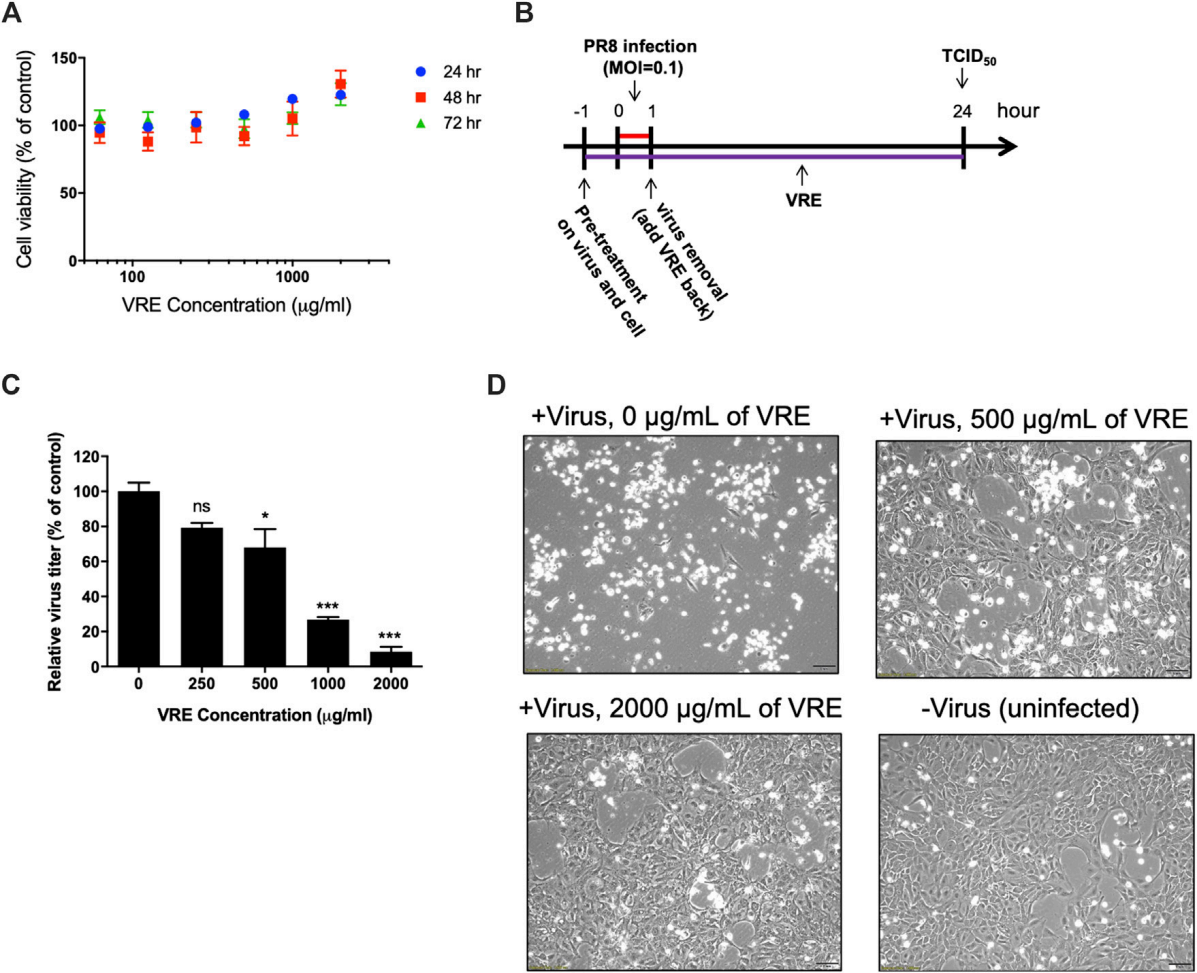
Cytotoxicity and virus inhibitory activity of VRE in MDCK cells. **(A)** MDCK cells were treated with various concentrations of VRE at 37°C for 24, 48, and 72 h. Cell viability was evaluated by MTT assays. Data in the plot present the mean ± SEM of four replicates. **(B)** Schematic diagram of the experimental design. PR8-H1N1 (MOI = 0.1) was treated with VRE at 37°C for 1 h. At the same time, MDCK cells were incubated with VRE in medium at 37°C for 1 h. After incubation, the VRE-virus mixture was used to infect cells for 1 h. The cells were washed after supernatant removal and then incubated with VRE for 24 h. The supernatants were collected for the TCID_50_ assay. **(C)** The viral titers of each group are indicated. Data in the plot present the mean ± standard error of the mean (SEM) of three replicates. Data were compared by one-way ANOVA followed by Dunnett’s multiple comparisons test (ns, non-significant; *p < 0.05, ***p < 0.001). **(D)** Images of MDCK cell were observed at 12 h post infection and compared with those of the uninfected control cells.

The cytotoxicity of VRE was evaluated in MTT assays. The results showed that VRE exhibited no cytotoxicity in MDCK cells, even at 2,000 μg/ml, the highest concentration used in this study (Figure 2A).

### *Vigna radiata* Extract Has Concentration-Dependent Anti-Influenza Virus Activity

The antiviral activity of VRE was demonstrated in a cellular infection model (Figure 2B). The results showed that 500, 1,000, and 2,000 μg/ml VRE significantly (*p* < 0.05) inhibited PR8-H1N1 virus infection by 68.0%, 26.9% and 8.5%, respectively, comparing with the vehicle control in MDCK cells, as determined by a TCID_50_ assay (Figure 2C). With images of uninfected cells as a reference, VRE treatment protected against the cytopathic effect induced by virus infection in a concentration-dependent manner (Figure 2D). These data suggested that VRE has potent antiviral activity against influenza virus.

### *Vigna radiata* Extract Exhibits Antiviral Activity in the Early and Late Stages of the Viral Life Cycle

**FIGURE 3:**
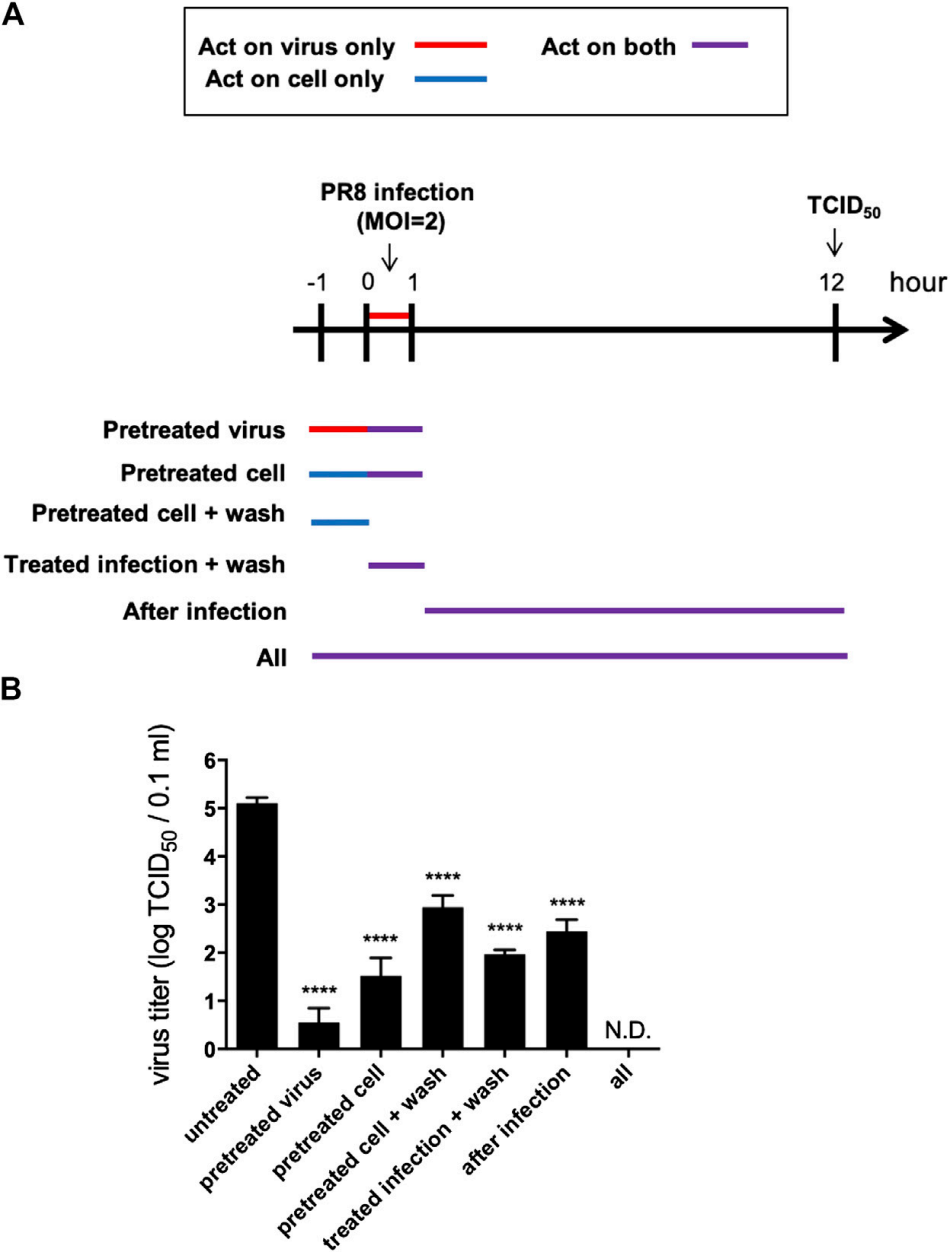
VRE interferes with the viral life cycle under different treatment conditions. **(A)** Schematic diagram of the experimental design. MDCK cells were infected with PR8-H1N1 (MOI = 2) under six different treatment conditions. At 12 hpi, the virus titers were determined by a TCID_50_ assay. **(B)** The viral titers of each group relative to the vehicle control are indicated. Data in the plot present the mean ± SEM of three replicates. Data were compared by one-way ANOVA followed by Dunnett’s multiple comparisons test (****p < 0.0001, N.D., not detectable).

To verify the antiviral mechanism of VRE, various time points during a single infectious cycle were used to determine the stages at which VRE exerted its inhibitory effect. MDCK cells were infected with PR8-H1N1 (MOI = 2) under six different treatment conditions: pretreatment of viruses and treatment during inoculation (pretreated virus), pretreatment of cells and treatment during inoculation (pretreated cells), pretreatment of cells (pretreated cells + wash), treatment during inoculation (treated infection + wash), treatment after inoculation (after infection), or all of the above time points (all) (Figure 3A). At 12 hpi, the antiviral activity was determined by a TCID_50_ assay. As shown in Figure 3B, “pretreated virus” significantly reduced virus titers (a reduction of 4.6 log_10_, *p* < 0.0001) compared to those of the vehicle control, suggesting that VRE may block viral HA or its host receptor, sialic acid, to interfere with the entry step. In line with this finding, “pretreated cells” and “pretreated cells + wash” significantly reduced virus titers (a reduction of 3.6 and 2.2 log_10_, respectively, *p* < 0.0001), indicating that VRE may block the cell surface receptor to inhibit viral entry. Similar results were found in the “treated infection + wash” group (a reduction of 3.2 log_10_, *p* < 0.0001). In the “after infection” group, VRE significantly decreased the virus titers (a reduction of 2.7 log_10_, *p* < 0.0001), suggesting that VRE may act on later stages of the viral life cycle, e.g., viral release. The “all” group showed complete elimination of virus infection, as VRE was able to act on the virus itself and on the host at multiple steps of viral replication in this experimental condition.

**FIGURE 4:**
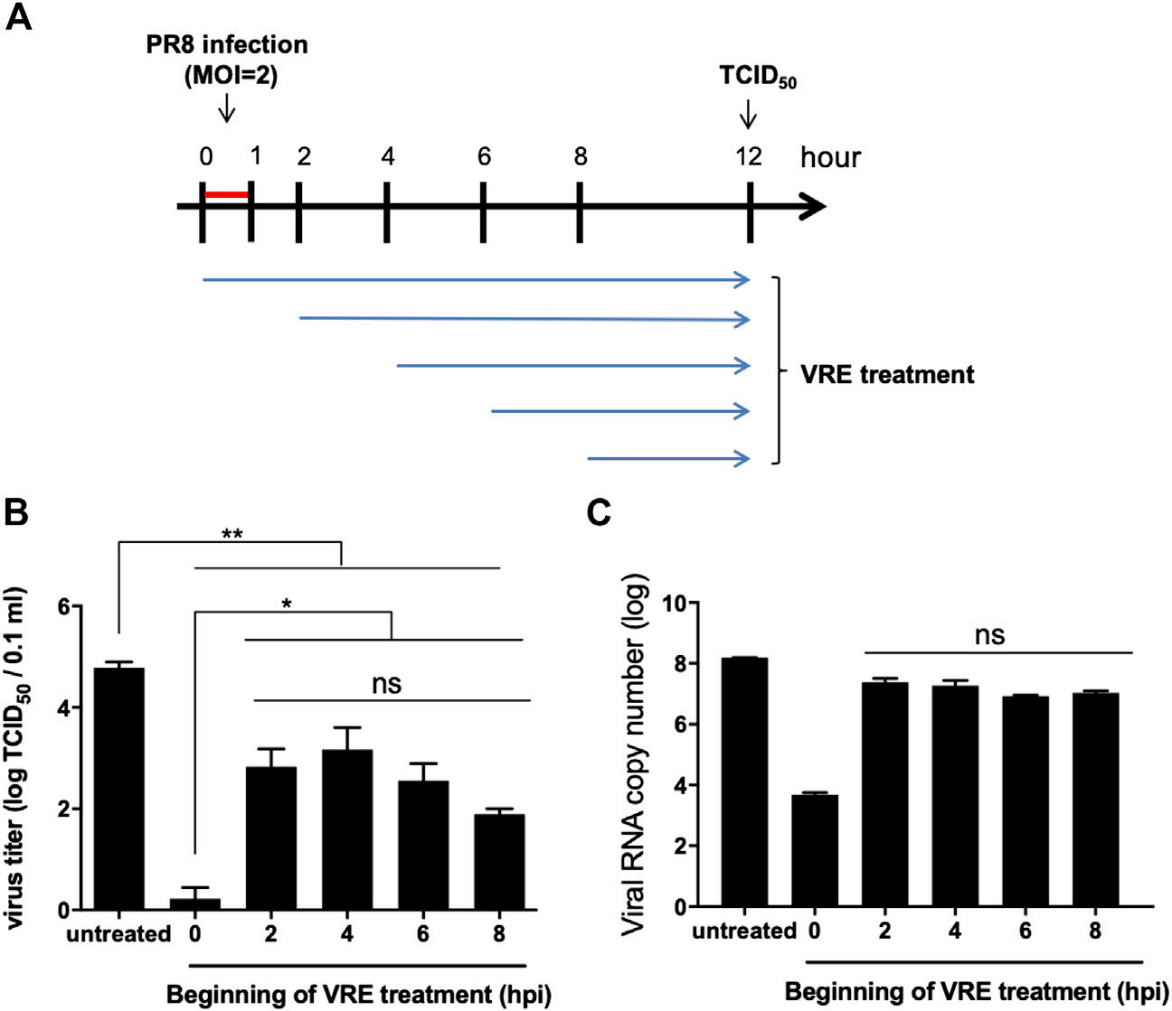
Virus inhibition by VRE treatment. **(A)** Schematic diagram of the experimental design. Different initial time points of VRE treatment of MDCK cells relative to PR8-H1N1 (MOI = 2) infection were tested to evaluate the viral inhibition effect of VRE. **(B)** At 12 hpi, the extracellular virus titers were determined by TCID_50_. **(C)** At 12 hpi, intracellular viral RNA was quantitated by qRT-PCR. The untreated cell group was used as a control. Data in the plot present the mean ± SEM of three replicates. Data were compared by one-way ANOVA followed by Dunnett’s multiple comparisons test (ns, non-significant; *p < 0.05, **p < 0.01).

Another addition assay was performed at five different times in a single infectious cycle to investigate which stage of the viral life cycle is inhibited by VRE (Figure 4A). The results indicated that VRE treatment at 8 hpi, the late stage of the infectious cycle, significantly reduced the virus titers (a reduction of 2.9 log_10_, *p* < 0.0001), suggesting that VRE could inhibit the viral release process (Figure 4B). However, VRE treatment at 2, 4, and 6 hpi had similar antiviral effects as treatment at 8 hpi, suggesting that VRE mainly inhibits viral release but does not interfere with other infectious stages after viral entry. Treatment at 0 hpi exhibited the greatest ability to inhibit viral replication, suggesting that VRE could also inhibit viral entry, which is consistent with the findings obtained for the “pretreated virus” and “pretreated cell” groups in Figure 3B. To further confirm the antiviral mechanism of VRE, intracellular viral RNA was detected in parallel, and the results showed that VRE treatment at 2, 4, 6, and 8 hpi affected viral RNA replication only slightly; in contrast, treatment at 0 hpi strongly decreased the viral RNA level (Figure 4C). Taken together, these data show that VRE mainly inhibits the viral entry and release processes.

### *Vigna radiata* Extract Blocks Both the Hemagglutinin Protein and Cellular Receptors

**FIGURE 5:**
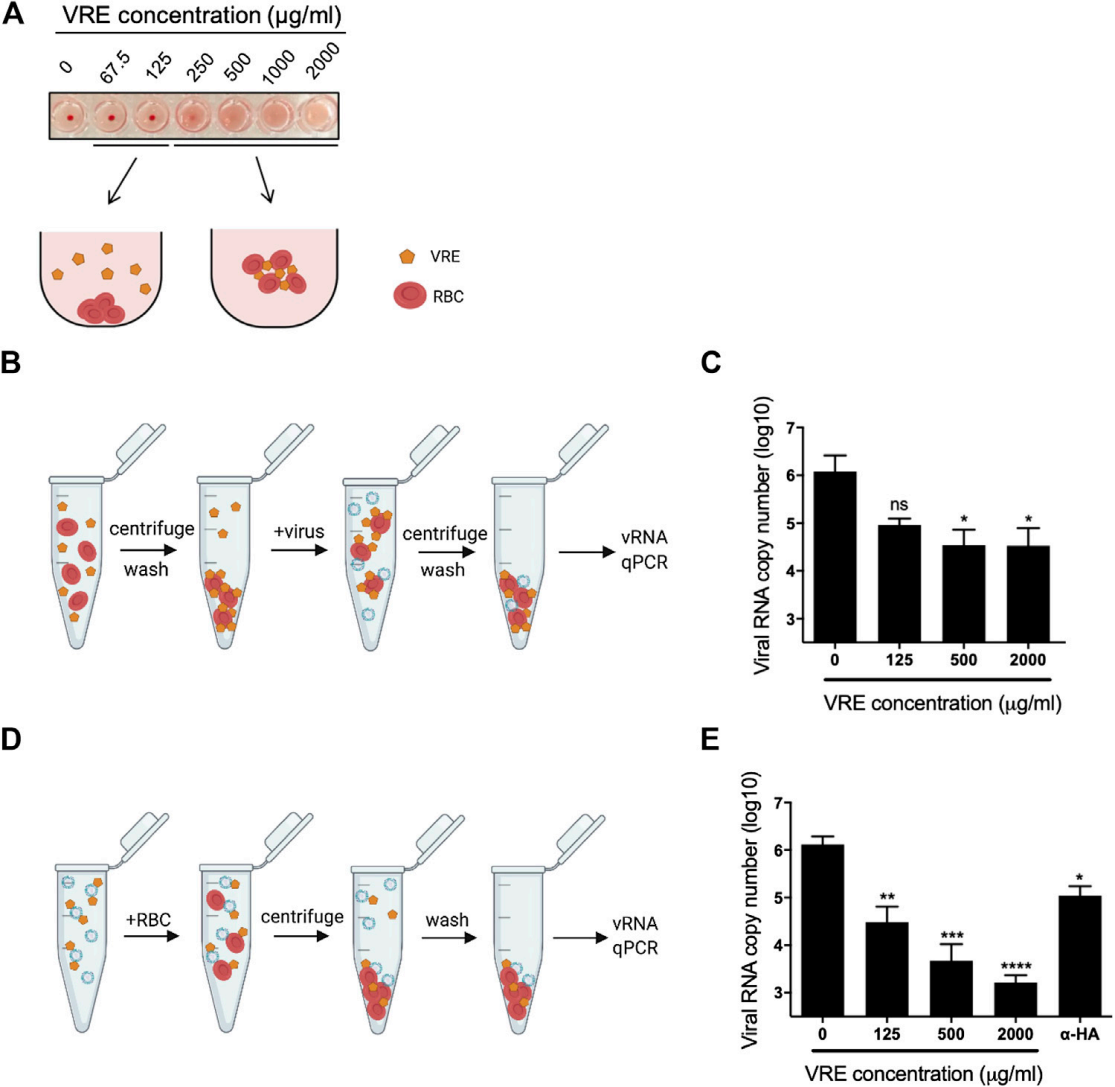
Hemagglutination assay using VRE-treated RBCs or viruses. **(A)** VRE-induced hemagglutination was illustrated. **(B)** Schematic diagram of the experimental procedures. RBCs were treated with various concentrations of VRE at RT for 30 min. The unbound VRE was removed, and the treated RBC pellet was washed and resuspended. A total of 2,000 HAU of PR8-H1N1 virus was added to the treated RBCs and incubated at RT for 30 min. Then, the RBC pellet was washed again and resuspended in PBS. **(C)** The amount of RBC-bound virus was determined by qRT-PCR. **(D)** Schematic diagram of the experimental procedures. A total of 2,000 HAU of PR8-H1N1 virus was treated with various concentrations of VRE or 2 μg of polyclonal antibody against the H1N1 HA protein at RT for 30 min. Then, 500 µl of 1% RBCs was added to the treated virus solution and incubated at RT for 30 min, and the RBC pellet was washed and resuspended in PBS. **(E)** The amount of RBC-bound virus was determined by qRT-PCR. Data are presented as the mean ± SEM of three replicates and compared by one-way ANOVA followed by Dunnett’s multiple comparisons test (ns, non-significant; *p < 0.05, **p < 0.01, ***p < 0.001, ****p < 0.0001).

To study the anti-entry activity of VRE, an RBC virus binding assay was performed to test whether VRE can directly block the viral HA protein or block cellular receptors. In the HA test of VRE, we found that VRE treatment at concentrations of 250 μg/ml and higher directly hemagglutinated chicken RBCs, indicating that VRE can interact with the sialic acid receptor on the RBC surface (Figure 5A). In the VRE pretreatment group (Figure 5B), VRE concentration-dependently inhibited virus-mediated RBC hemagglutination, suggesting that VRE could block cell receptors (Figure 5C). Next, to dissect the ability of VRE to bind to the viral HA protein, hemagglutination was performed using the VRE-treated virus (Figure 5D). In the VRE pretreatment group, similar to the anti-hemagglutination activity induced by the anti-HA antibody, VRE inhibited virus binding with RBCs in a concentration-dependent manner, suggesting that VRE is able to block the viral HA protein (Figure 5E).

### *Vigna radiata* Extract Inhibits Viral Penetration

**FIGURE 6:**
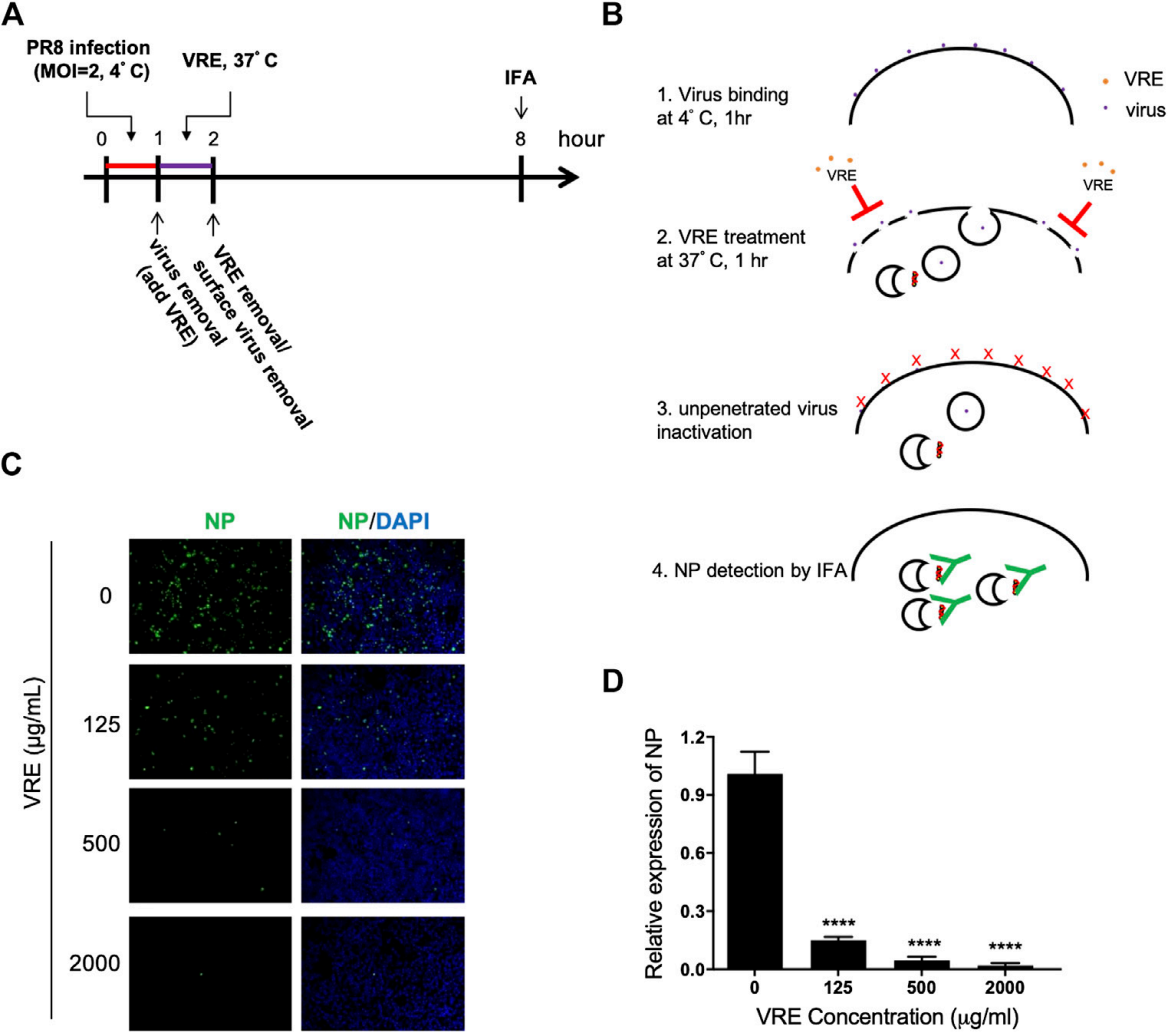
VRE blocks virus penetration. **(A,B)** Schematic diagram of the experimental procedures. **(C)** MDCK cells were infected with PR8-H1N1 (MOI = 2) at 4°C for 1 h for virus attachment. One hour later, VRE was applied to the cells for 1 h to inhibit virus penetration. The unpenetrated virus was inactivated with PBS at pH 2 for 1 min and neutralized with PBS at pH 11. The cells were then incubated in fresh medium for an additional 6 h. The virus signal was detected via immunofluorescence staining with an NP antibody. **(D)** The fluorescence intensity was measured with a microplate reader. Relative NP expression levels were determined. Data are presented as the mean ± SEM of three replicates and were compared by one-way ANOVA followed by Dunnett’s multiple comparisons test (****p < 0.0001).

We next tested whether VRE can interfere with the penetration process. Virus was first absorbed on the cell surface before treatment with VRE. The penetrated virus was detected via immunofluorescence (Figures 6A,B). Viral NP expression was decreased in the presence of VRE treatment (Figure 6C), indicating that VRE inhibited viral penetration in a concentration-dependent manner (Figure 6D). Further studies are warranted to investigate the detailed mechanism by which VRE interferes with endocytosis, endosomal acidification or membrane fusion.

### *Vigna radiata* Extract Inhibits *α*-Glucosidase Activity and Viral Assembly

**FIGURE 7:**
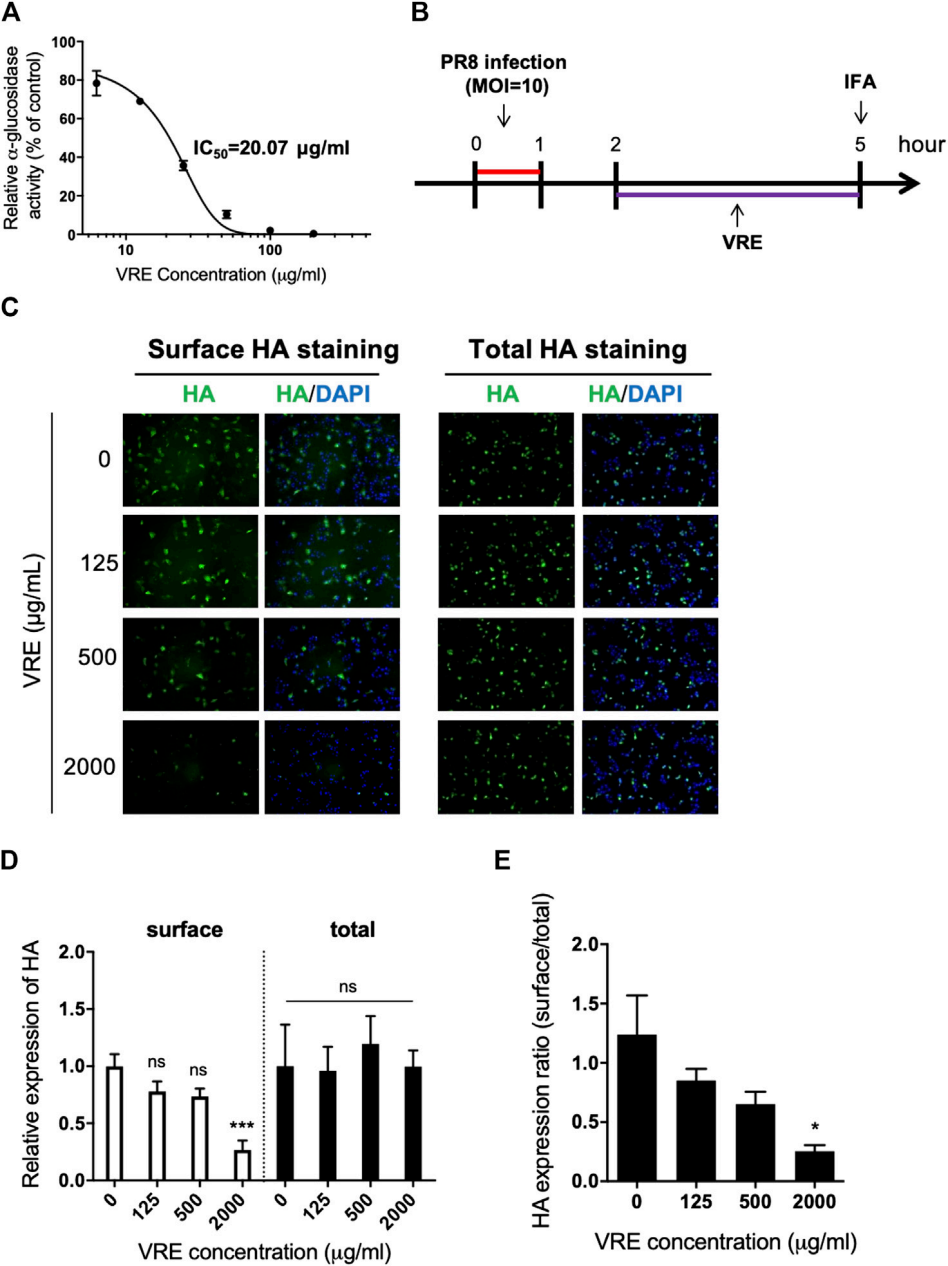
VRE inhibits *α*-glucosidase activity in a concentration-dependent manner and interferes with the surface expression of HA. **(A)** *α*-Glucosidase activity was measured by detecting the absorbance at 450 nm and normalized to the control. The IC_50_ of VRE in inhibiting *α*-glucosidase is indicated. **(B)** Schematic diagram of the experimental procedures. MDCK cells were infected with PR8-H1N1 (MOI = 10) and then treated with various concentrations of VRE at 2–5 h post infection. The cells were then washed and fixed with or without permeabilization. HA expression was detected via immunofluorescence staining with an HA antibody. **(C)** HA expression on the surface (left, cells were fixed only) or in the entire cell (right, cells were fixed and permeabilized) was imaged and merged with DAPI-stained images. **(D)** Fluorescence intensity was measured. The HA expression levels relative to the nuclear signal were determined from the two different types of staining methods. **(E)** The ratio of surface/total HA expression under various VRE treatments is shown. Data are presented as the mean ± SEM of three replicates and compared by one-way ANOVA followed by Dunnett’s multiple comparisons test (ns, non-significant; *p < 0.05, ***p < 0.001).

It has been reported that inhibition of *α*-glucosidase leads to a reduction in viral assembly and release because the viral glycoprotein cannot be modified before transport to the cell membrane. Therefore, we sought to investigate whether VRE has anti-α-glucosidase activity. VRE inhibited *α*-glucosidase activity in a concentration-dependent manner with an IC_50_ of 20.07 μg/ml (Figure 7A). We further investigated viral HA expression in MDCK cells in the presence of VRE (Figure 7B). In line with the observed enzyme-inhibiting activity, cells treated with VRE exhibited less HA expression on the cell surface than did the cells in the untreated group; in contrast, the amount of total intracellular HA expression remained unaffected (Figures 7C,D). The ratio of surface HA to total intracellular HA was calculated, and treatment with 2,000 μg/ml VRE resulted in a significant difference (Figure 7E). These results indicated that VRE can affect glycoprotein modification and trafficking onto the cell surface and thus inhibit viral assembly.

### *Vigna radiata* Extract Inhibits Neuraminidase Activity and Viral Release

**FIGURE 8:**
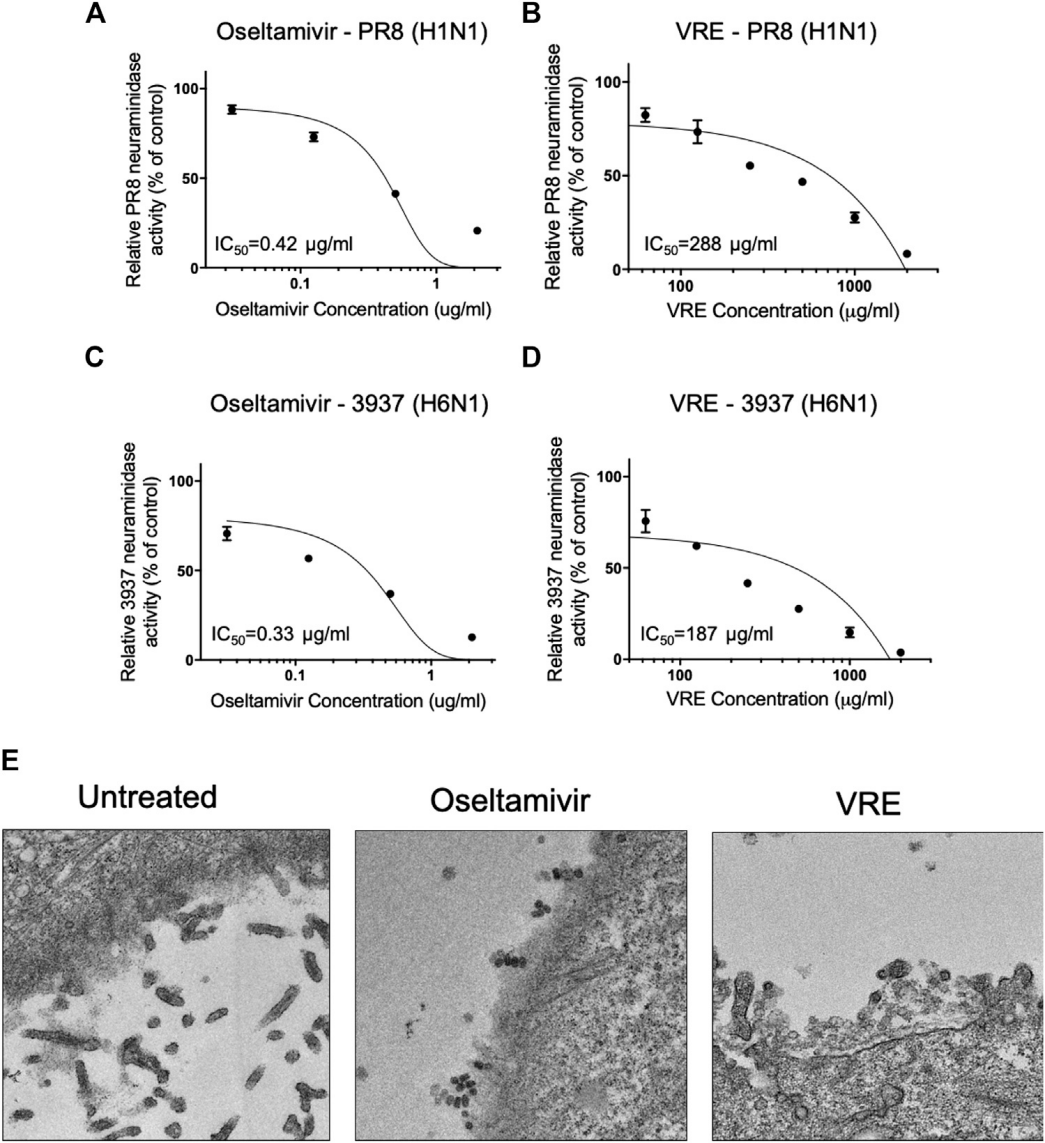
VRE inhibits viral neuraminidase activity in a concentration-dependent manner. The PR8-H1N1 neuraminidase inhibition activity of oseltamivir **(A)** and VRE **(B)** was measured by detecting fluorescence and normalized to the control. The 3937-H6N1 neuraminidase inhibition activity of oseltamivir **(C)** and VRE **(D)** was measured by detecting fluorescence and normalized to the control. The IC_50_ for inhibiting neuraminidase is indicated. Data are presented as the mean ± SEM of three replicates. **(E)** MDCK cells were infected with PR8-H1N1 (MOI = 5) for 1 h, and the cells were treated with VRE (2,000 μg/ml) or oseltamivir (5 μg/ml) for 6–12 h post infection. TEM images demonstrated different levels of virus release from infected MDCK cells.

To determine the antiviral release-associated mechanisms of VRE, we tested whether VRE inhibits viral neuraminidase. Neuraminidase from the mammalian influenza virus PR8-H1N1 and the avian influenza virus 3937-H6N1 were utilized to investigate the inhibition ability of VRE. As shown in Figures 8A,B, similar to the well-known neuraminidase inhibitor oseltamivir, which showed a low IC_50_ of 0.42 μg/ml, VRE inhibited the neuraminidase activity of the H1N1 virus in a concentration-dependent manner and had an IC_50_ of 288 μg/ml. In Figures 8C,D, while oseltamivir showed an IC_50_ of 0.33 μg/ml in inhibiting the neuraminidase activity of 3937-H6N1, an avian influenza strain, VRE also effectively inhibited the neuraminidase activity of the 3937-H6N1 virus, with an IC_50_ of 187 μg/ml. Furthermore, TEM imaging clearly showed that treatment of MDCK cells with 2,000 μg/ml VRE blocked virus release from infected cells, similar to what was observed in the oseltamivir-treated group (Figure 8E).

## Discussion

In this study, VRE’s antiviral activity against the influenza virus was discovered. We confirmed that VRE protected cells from influenza virus-induced cytopathic effects and significantly inhibited viral replication, with effects on both the mammalian H1N1 and avian H6N1 viruses, in a concentration-dependent manner. In addition, VRE was safe in cells. Until 72 h of incubation, VRE showed no toxicity in cells, indicating that VRE has potential for development into a safe and effective pharmaceutical compound.

The detailed time-of-addition assay revealed that VRE may act on both the early and late stages of the viral life cycle. Therefore, we further dissected the role of VRE in inhibiting viral entry. For influenza virus, the HA protein is the entry determinant and can agglutinate RBCs; this process is known to be mediated by crosslinking sialic acid on the surface of RBCs. From the RBC masking assay, we showed that VRE is able to trigger RBC agglutination and mask the cell surface in a concentration-dependent manner, thus preventing the subsequent attachment of influenza virus. However, whether VRE acts directly on the viral receptor, namely, sialic acid, or on other molecules and influences receptor activity through steric hindrance remains to be explored. On the other hand, in the hemagglutination-inhibition test, we also showed that VRE directly attached to the virion and blocked the subsequent attachment of the virus to RBCs. More importantly, similar inhibition of virus binding could be further validated in susceptible cells. In light of its dual mechanisms of action in blocking viral entry, VRE shows prominent antiviral activity in the early stage. Furthermore, as VRE is able to mask the RBC surface, similar inhibition of binding for other RBC-agglutinating viruses, such as adenoviruses, paramyxoviruses and coronaviruses, is possible and should be investigated in future studies.

Endosomal delivery of influenza virus is one of the critical steps in establishing viral infection and can become an attractive target mechanism for antivirals. In this study, after successful binding of the virus to cellular receptors, VRE was shown to influence the cellular endocytosis machinery and viral uncoating/fusion, thereby achieving significant antiviral activity. Other antiviral drugs involved in inhibiting endosomal acidification include vacuolar ATPase blockers (Chen et al., 2013), chloroquine (Yan et al., 2013), and fusion inhibitors (Kadam and Wilson, 2017). These drugs have demonstrated broad-spectrum activity against multiple types of viruses, as many viruses adopt similar endocytosis-dependent infection processes.

Considering the known anti-α-glucosidase activity of vitexin and isovitexin (He et al., 2016), two active components of VRE, we first confirmed that VRE showed anti-α-glucosidase activity, although it had a higher IC_50_ (20.07 μg/ml) than the pure compounds of vitexin and isovitexin. Then, we demonstrated that VRE significantly influences HA protein surface presentation in a concentration-dependent manner. *α*-Glucosidase plays a pivotal role in the glycosylation process of several biological molecules, including viral surface proteins. Previous studies have shown that altering the glycosylation of the HA protein may severely impact viral infectivity. To our knowledge, this is the first report of an influenza-inhibiting mechanism mediated by anti-α-glucosidase activity in a natural product.

Previous studies have shown that flavonoids such as apigenin, a flavonoid commonly found in natural plants, have neuraminidase-inhibiting activity (Liu et al., 2008). In a docking study, vitexin was shown to have binding affinity with influenza A virus neuraminidase (Sadati et al., 2019). Similarly, the anti-influenza A virus H1N1 ability of isovitexin, with an IC_50_ of 14 μg/ml, has been reported (Ye et al., 2011), and the potential mechanism is through the viral neuraminidase inhibition (Luo et al., 2020). In this study, we confirmed that VRE showed anti-neuraminidase activity against two different subtypes of influenza virus. Nevertheless, the IC_50_ of VRE is far higher than that of oseltamivir, a pure neuraminidase inhibitor (Bassetti et al., 2019). While many identified influenza viruses exhibit anti-oseltamivir resistance, VRE’s neuraminidase-inhibitory activity deserves further study.

In summary, VRE potently interferes with influenza virus infection at multiple steps during the infectious cycle (Supplementary Figure S1), demonstrating its broad-spectrum potential as an anti-influenza prevention and treatment agent. Continued development of VRE to generate antiviral therapeutics is warranted.

## Data Availability Statement

The raw data supporting the conclusions of this article will be made available by the authors, without undue reservation, to any qualified researcher.

## Author Contributions

H-WC conceived and designed the experiments. C-WL, C-CP, and Y-TC performed the experiments and analyzed the data. C-WL drafted the manuscript. H-WC edited the manuscript. All authors read and approved the final manuscript.

## Funding

This work was supported by King’s Ground Biotech Co., Ltd. (06HT617007 to H-WC) and National Taiwan University. The funders had no role in the study design, data collection and analysis, decision to publish, or preparation of the manuscript.

## Conflict of Interest

C-CP is a current employee of King’s Ground Biotech Co., Ltd. This does not alter our adherence to the policies set forth by the journal on data and material sharing.

The remaining authors declare that the research was conducted in the absence of any commercial or financial relationships that could be construed as a potential conflict of interest.
