# Supplementary material for: Vigna radiata (L.) R. Wilczek Extract Inhibits Influenza A Virus by Targeting Viral Attachment, Penetration, Assembly, and Release
Source: Front Pharmacol. 2020 Nov 26;11:584973. doi: 10.3389/fphar.2020.584973 (PMC7725899; doi:10.3389/fphar.2020.584973)
Supplement: Supplementary file 1 [file presentation1.pptx]

## Slide 1
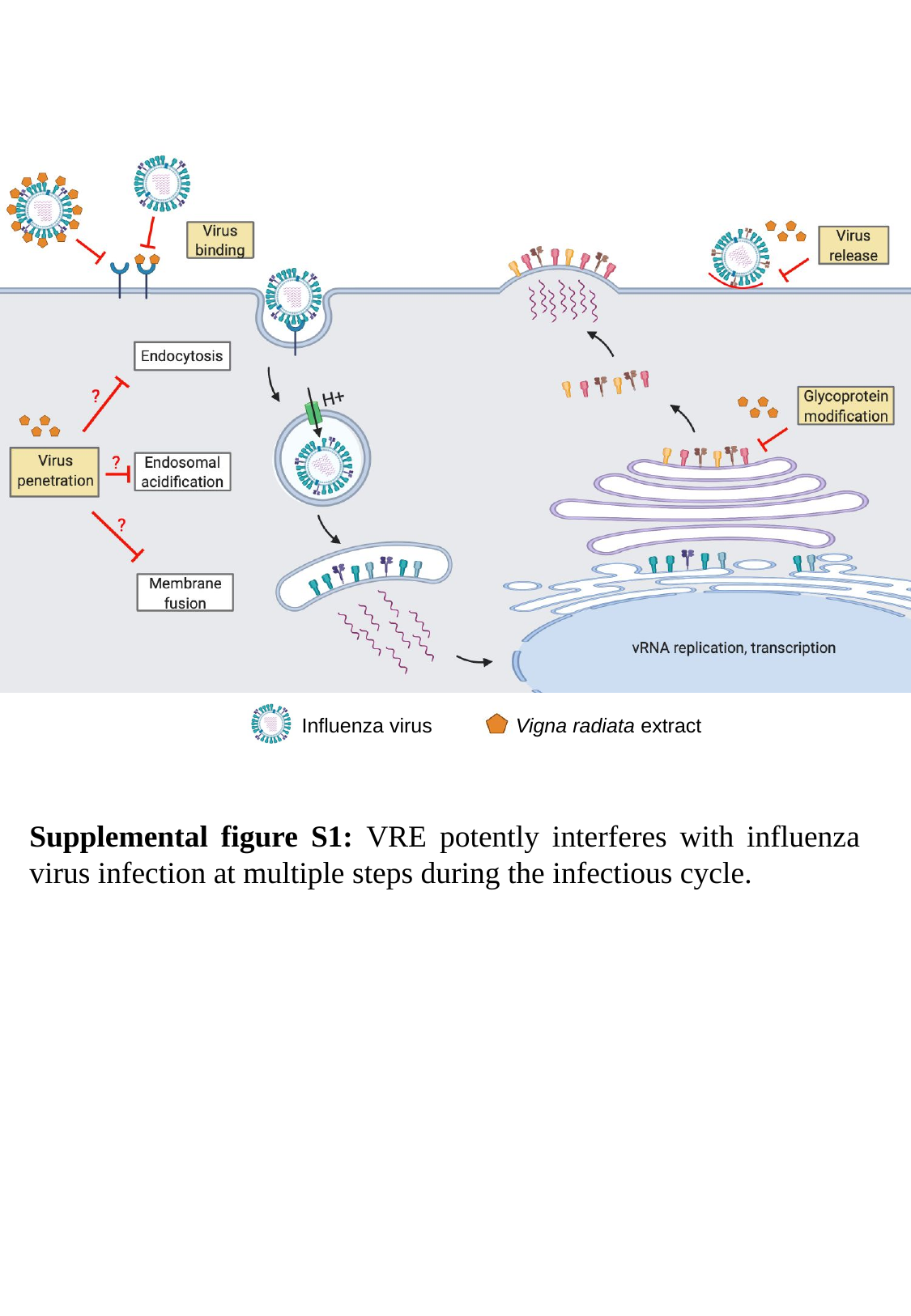

Vigna radiata extract
Influenza virus
Supplemental figure S1: VRE potently interferes with influenza virus infection at multiple steps during the infectious cycle.
